# Ethosomal gel for rectal transmucosal delivery of domperidone: design of experiment, *in vitro,* and *in vivo* evaluation

**DOI:** 10.1080/10717544.2022.2072542

**Published:** 2022-05-11

**Authors:** Wedad Sakran, Rania S. Abdel-Rashid, Fatma Saleh, Raghda Abdel-Monem

**Affiliations:** Department of Pharmaceutics and Industrial Pharmacy, Faculty of Pharmacy, Helwan University, Cairo, Egypt

**Keywords:** Domperidone, nanovesicles, ethosomes, full factorial design, rectal transmucosal delivery

## Abstract

Despite high efficiency of domperidone (DOM) in prophylaxis of emesis accompanied with radiotherapy and chemotherapy, it still can bother cancer patients by its powerful side effects and difficulty of its oral administration. The study was designed to develop and optimize DOM loaded ethosomal gel for rectal transmucosal delivery. Ethosomal formulations were prepared using a 2^1^, 5^1^ full-factorial design where the impact of lecithin concentration and additives were investigated. The optimum ethosomal vesicles were subsequently incorporated in Carbopol gel base where rheological behavior, spreadability, mucoadhesion, and *in vivo* pharmacokinetic parameters were studied. Based on Design Expert^®^ software (Stat Ease, Inc., Minneapolis, MN), the optimum formulation illustrated entrapment efficiency of 70.02%±5.52%, and vesicular size of 112 ± 3.3 nm, polydispersity index of 0.32 ± 0.01, zeta potential of −59 ± 0.28 mV, and % drug released after 6 h of 76.30%±2.45%. Moreover, *ex vivo* permeation through rabbit intestinal mucosa increased four times compared to free DOM suspension. The gel loaded with ethosomes showed excellent mucoadhesion to rectal mucosa. DOM ethosomal gel showed a raise in *C*_max_ and AUC_0–48_ of DOM by twofolds compared to free DOM gel. The study suggested that ethosomes incorporated in gels could be an efficient candidate for rectal transmucosal delivery of DOM.

## 1. Introduction

Side effects of anti-cancer drugs are often more troubling than the actual symptoms of cancer disease for many patients. Nausea and emesis are the most frequent, stressful, and feared side effects among patients receiving chemotherapy. They can affect patients’ quality of life and their ability to endure and comply with therapy (Matthews et al., 2015). Therefore, suppression of emesis is a dream that cancer patients wish to fulfill and improve the quality of their lives (Satija et al., 2014). For this purpose, dopamine antagonist antiemetic’s such as Domperidone (DOM), bromopride, and metoclopramide have been widely administered via parenteral or oral routes on daily basis (Hesketh et al., 2017). Domperidone, a selective peripheral dopamine antagonist, that boosts natural gastric activity, raises the gastric emptying rate (Patil et al., 2016), enhances the power of duodenal contractions, and drops small bowel transit time. It is commonly used for the short-term treatment of nausea and emesis correlated with cancer chemotherapy. Moreover, it is considered as an adjuvant therapy in the management of Parkinsonism, and disorders of gastrointestinal motility (Ferrier, 2014). Several reports indicated that DOM has shown more statistically significant therapeutic efficiency than ondansetron at the same dose for the prevention of late gastro-intestinal disturbance symptoms occurring after highly emetogenic chemotherapy (Phillips et al., 2016). However, DOM has poor aqueous solubility (1 mg/mL) and low systemic bioavailability (13–17%) due to broad first pass metabolism in the stomach wall and liver (Athukuri & Neerati, 2017).

Hence, the delivery of antiemetic drugs via transmucosal route is a smart pathway for retaining constant blood concentration by making drugs directly exert their systemic effect avoiding first pass metabolism associated with oral administration. Additionally, it seems to recover medication compliance in patients. Moreover, it might also be useful for both pediatric and geriatric patients experiencing nausea and emesis (Kadam et al., 2017). Lipidic nanocarriers such as, liposomes are phospholipid vesicles which consist of single or more lipid bilayers enveloping aqueous core; although they are considered a potential solution for development of new delivery systems, they mostly cannot break through the systemic circulation (El-Menshawe et al., 2017). Therefore, innovative forms of lipid-based vesicles as transfersomes and ethosomes have been developed (Song et al., 2012). Transfersomes which were introduced by Cevc & Blume (1992) were considered to be the original form of highly penetrative flexible lipid vesicles produced (Bendas & Tadros, 2007). They could be described as lipid-based nanovesicles that are mainly composed of edge activators that disrupt the lipid bilayer and hence increase the flexibility and penetration (Honeywell-Nguyen et al., 2002). Among recent vesicular systems, ethosomes were selected as they possess many advantages. Ethosomes differ from liposomes by its high concentration of ethanol, in addition to phospholipid and water which enhance its permeation by increasing the fluidity of membranes (El Sayed et al., 2006). The high elasticity of ethosomal nanovesicles was resulted from the added ethanol which allows them to squeeze and penetrate, consequently enhance efficient drug delivery. Moreover, ethanol increase solubility of both hydrophilic and lipophilic drugs permits their entrapment in the entire vesicle (Verma & Pathak, 2012). These classical ethosomes can be modified by introducing other compounds to improve vesicular characterization and tissue permeation, such as binary ethosomes; developed by inserting different kind of alcohol to the classical ethosomes such as propylene glycol (PG) (Zhou et al., 2010). Propylene glycol is the widely used alcohol in ethosomes which act as penetration enhancer (Shen et al., 2014). Furthermore, transethosomes (TEs) provided certain modification for ethosomal systems by incorporating an edge activator added to the basic components of classical ethosomes (Song et al., 2012). These new lipid-based nanovesicles (TEs) were developed to merge the advantages of both classical ethosomes and transfersomes in one formula by combining liquid lipid (e.g. oleic acid and Labrafac^®^) with solid phospholipid. They were reported to increase the drug solubility and entrapment efficiency by introducing enough space to accommodate drug molecules in the crystal lattice resulted from incorporation of liquid lipid to solid lipid (Radtke et al., 2005).

Incorporation of ethosomes into hydrogels was suggested by El-Menshawe et al. (2017) who reported the effect propranolol HCL ethosomal gel in drug delivery through buccal mucous membrane showed high capability of ethosomal gel in drug delivery and enhancing bioavailability (El-Menshawe et al., 2017). Incorporation of ethosomes into gel could also enrich their stability and transmucosal penetration ability. This returned to the great compatibility between ethosomal carriers and hydrogels afford better bioadhesive properties rendering satisfactory permeation for transmucosal drug delivery (Rao et al., 2021).

Therefore, the study aimed to utilize full factorial design (2^1^, 5^1^) to investigate the effect of formulation variables (phospholipid concentration, type of additives) as independent variables on physicochemical characterization of ethosomal suspensions (vesicle size, zeta potential (ZP), % entrapment efficiency, and *in vitro* drug release) as dependent variables. Design of experiment (DoE) approach was used for analyzing the data statistically and graphically using response surface plots. Besides, the optimized ethosomal suspension was loaded into hydrogel that was subsequently applied over rectal mucosa. DOM loaded ethosomal gels were characterized as topical preparation for their physical assets, rheological behavior, and mucoadhesive strength. An *in vivo* model was conducted to study pharmacokinetic parameters in comparison to conventional free drug loaded gels.

## 2. Materials and methods

### 2.1. Materials

Domperidone was generously donated from Sedico Pharmaceutical Company (6th of October City, Egypt). Lecithin was given as a gift from NODCAR. Ethanol, methanol, acetonitrile, and formic acid (HPLC grade) were obtained from Sigma-Aldrich Chemical Co. (St Louis, MO). Propylene glycol dicaprylocaprate (Labrafac^®^) was kindly gifted by Gattefosse (Saint-Priest, France). Carbopol 934 was bought from BF Goodrich (Akron, OH). Sodium dihydrogen phosphate, disodium hydrogen phosphate, and triethanolamine (TEA) (analytical grade) were purchased from El-Gomhouria Chemicals Pharmaceutical Company (Cairo, Egypt). Analytical grade of glycerin, PG, tween 80, span 60, and oleic acid were attained from El-Nasr Pharmaceutical Chemicals Co. (Cairo, Egypt).

### 2.2. Animals

Male rabbits (2–3 kg ± 0.5 kg, 2 months old) and adult male Wistar rats (190–210 g, 2 months old) were supplied by Experimental Animal Center of Faculty of Pharmacy, Helwan University (Cairo, Egypt). All the animals were kept and used for the experiment in accordance with the Animal Research Ethical Committee, animal experiments of Pharmaceutical Sciences Faculty of Pharmacy, Helwan University (code 03A2021).

### 2.3. Experimental design

A complete 2^1^, 5^1^ multi-level factorial design was created by Design Expert^®^ software, version 12.0 (Stat Ease, Inc., Minneapolis, MN) to determine the effect of different variables on DOM-loaded ethosomal suspensions using the low experimental runs. In this chosen design, the effects of two independent variables specifically, lecithin concentration (1st factor) at two levels (2% and 3%) and type of additives (2nd factor) at five levels (none, oil A (Labrafac^®^), oil B (oleic acid), tween 80, and span 60) on five responses as namely, vesicular size (PS), polydispersity index (PDI), electrical double layer ZP, % efficiency of drug entrapment (%EE), and % cumulative drug release after 6 h (Q6h) were estimated (Table 1). The experimental design covered all probable formulations for incorporation of DOM-loaded into ethosomal suspensions as shown in Table 2. The experimental data were evaluated for significance by the analysis of variance (ANOVA) test using Design Expert^®^ software (Stat Ease, Inc., Minneapolis, MN). Desirability was calculated for selection of optimum formulation after insertion of desired outcomes. The criterion set for deciding the optimized formulation was accomplishment of the least PS, PDI and the highest %EE, ZP, and Q6h.

**Table 1.** Full factorial design (2^1^, 5^1^) used for optimization formulations showing independent, dependent variables (responses) and desired outcomes.

Independent variables	Levels	
	Low	High
Lecithin concentration	2%	3%
Additives	None	Tween 80, Labrafac^®^, oleic acid, span 60

Dependent variables	Unit	Goal
Mean vesicular size	nm	Minimize
PDI	–	Minimize
Zeta potential	mV	Maximize
EE	Percent	Maximize
Q6h	Percent	Maximize

PDI: polydispersity index; EE: entrapment efficiency; Q6h: % cumulative drug release for 6 h.

**Table 2.** Composition of DOM-loaded ethosomal suspensions.

Formulation code	Total lipid content (lecithin/oils) (%)	Additives			
		Tween 80 (% w/v)	Span 60 (% w/v)	Labrafac^®^ (% w/w of total lipid)	Oleic acid (%w/w of total lipid)
F1	2	–	–	–	–
F2	2	–	–	–	10 %
F3	2	–	–	10%	–
F4	2	2%		–	–
F5	2		2%		
F6	3	–	–	–	–
F7	3	–	–	–	10%
F8	3	–	–	10%	–
F9	3	2%	–	–	–
F10	3	–	2%	–	–

Each formula also contains 30% ethanol, 10 mg DOM, and 1 mL PG.

### 2.4. DOM loaded ethosomes

Ethosomal dispersions encapsulating DOM were formulated according to the method developed by Touitou et al. (2001) with modification as it is simple and most popular method used in preparing ethosomal systems. The organic and aqueous phases were prepared separately. The organic phase containing lecithin and DOM dissolved in ethanol at room temperature under vigorous stirring (1200 rpm) by means of magnetic stirrer. PG followed by water was then added dropwise using syringe and the mixture was agitated at 700 rpm for 30 min to produce the requisite ethosomal suspension (El-Shenawy et al., 2019). The effect of replacing 10% of lecithin concentration with oleic acid or Labrafac^®^ was studied. Addition of 2% (w/v) surfactant solution (span 60 and tween 80) to the aqueous or organic phase, respectively, was investigated too. The obtained suspension was sonicated using probe sonicator ultrasonic processor (UP50H, Hielscher, Teltow, Germany) with frequency 20 kHz at 4 °C for 5 min. Formulations were stored in the refrigerator and evaluated for vesicular size, ZP, %EE, and Q6h. Table 2 displays composition of different formulations based on the different screened variables.

### 2.5. Characterization of DOM loaded ethosomes

#### 2.5.1. Determination of PS and zeta potential

The ethosomal nanocarriers were analyzed for their PS and PDI using dynamic light scattering (DLS) technique. Laser Doppler velocimetry (LDV) was utilized for zeta-potential measurement. The equipment used for this study was Zeta-sizer Nano ZS (Malvern Instrument, Malvern, UK) with disposal cuvette for P.S. and cuvette DTS-1060 for zeta potential. Double-distilled and deionized water was applied through the study to prevent any kind of charge fluctuation and multi-scattering. Each sample was diluted 20 times with double distilled water prior measurement. Three measurements were performed for each sample at an angle of 90° at room temperature (25 °C) (Abdellatif & Tawfeek, 2016).

#### 2.5.2. Efficiency of drug entrapment

First, maximum wavelength (*λ*_max_) for pure DOM was determined by scanning solution of DOM in pure state in UV range 200–400 nm against blank. The accuracy of UV-spectrophotometric method was investigated by comparing estimation of DOM at the same concentration using HPLC. The %mean recovery of drug in pure state at different concentrations was around 99%. The low values of SD and %RSD (<2%) confirm high precision and accuracy of the proposed method. A specific volume of DOM loaded ethosomes was diluted with ethanol (1:10, v/v) and filtered with nylon syringe filter (0.22 µm pores) then analyzed using UV spectrophotometer at *λ*_max_ 284 nm against empty ethosomal suspension as a blank. Ultracentrifugation method was used to determine %EE where free (unentrapped) DOM was separated from ethosomal suspension utilizing a cooling centrifuge (Sigma Laborzentrifugen, Osterode am Harz, Germany) at 4 °C and 20,000 rpm for 20 min (Sguizzato et al., 2020). The supernatant which is suggested to be containing the unentrapped drug was diluted with certain amount of ethanol followed by measuring using UV spectrophotometer (Perkin Elmer UV, Yokohama, Japan) at 284 nm. The % of EE of the vesicles was then calculated, using the following equation (Sayed et al., 2015):

$$\%\mathrm{EE}=\frac{\text{total amount of drug}-\text{amount of unentrapped drug }}{\text{total amount of drug}}\times 100$$

#### 2.5.3. *In vitro* drug release (determination of Q6h)

The effect of formulation variables on DOM released from ethosomes was investigated using USP dissolution apparatus (Hanson Research Dissolution Tester, Chatsworth, CA) at body temperature 37 °C. Samples of 5 mL ethosomal suspension were located in cylindrical plastic tubes with a specific area of 2.2 cm^2^. One end of the tube was tightly wrapped with cellulose membrane and the other end was fixed to the shaft of the USP dissolution apparatus instead of the baskets. The formulae were dipped in 500 mL PBS (pH 6.8). The sink conditions were considered along the study (Safwat et al., 2017). As previously published after 1, 2, 3, 4, 5, and 6 h certain volumes were withdrawn and substituted by equal volume of fresh medium to maintain sink conditions (Albash et al., 2019). The cumulative amounts of DOM released from ethosomes were analyzed spectrophotometrically at *λ*_max_ 284 nm. The experiment was repeated for three times.

### 2.6. Physicochemical characterization of optimum ethosomal suspension

According to the results of Design Expert^®^ (Stat Ease, Inc., Minneapolis, MN) and based on the data obtained from experiments in previous section, the most desirable formulation was deducted. The optimum formulation was subsequently investigated.

#### 2.6.1. Transmission electron microscopy (TEM)

The morphology of the optimum formulation was examined using TEM (JEOL, JEM, Tokyo, Japan). A globule of freshly prepared ethosomal suspension was deposited onto the surface of carbon coated copper grid; natively dyed by phosphotungstic acid 1.5% and dried at room temperature for 15 min. The stained sample was then probed and visualized using TEM. Photographs were taken at suitable magnifications (Mukherjee et al., 2009).

#### 2.6.2. Differential scanning calorimetry (DSC)

The thermal analysis of pure DOM, lecithin, and optimum formula were accomplished using a thermal analysis system (DSC-40, Shimadzu, Kyoto, Japan) standardized with purified indium. Accurately weighed 5 mg of each sample was captured and sealed in flat bottomed aluminum pan with crimped on lid. The samples were subjected to heat at 5 °C/min rate under inert nitrogen gas atmosphere over temperature ranging from 10 to 400 °C. A similar empty pan was used as a reference.

#### 2.6.3. Fourier-transform infrared (FTIR) spectroscopy

FTIR is performed to determine a possible interaction between pure drug, excipients and the optimum formulation. The IR spectra of DOM, PC, and optimum formula F3 were recorded using an IR-Spectrophotometer (IR affinity-1, Shimadzu, Kyoto, Japan) between 400 and 4000 cm^−1^ to detect any drug–excipient interactions.

#### 2.6.4. *Ex vivo* transmucosal permeation

The permeation profile of DOM from selected ethosomal suspension through rabbit intestinal mucosal tissue was estimated (Abdellatif et al., 2017). The experimental protocol was approved by the Animal Research Ethical Committee, Faculty of Pharmacy, Helwan University (code 03A2021). A rabbit (weighing 2 kg ± 10%) was fasted for 12 h, then anesthetized and incised to isolate intestinal tissue. Equal segment sacs of rabbit intestinal mucosal tissue were thoroughly washed with Tyrode’s solution to remove any lumen contents. Each segment sac was tied from both ends after being filled with 5 mL of ethosomal suspension included calculated amount of DOM. The tissues were fixed in aerated organ baths at 37 °C where the receptor cell was filled with 40 mL of PBS (pH 6.8) (Organ Bath, RUMO, Cairo, Egypt). The experiment was carried out for 24 h under constant stirring rate. At precise time intervals, a specific volume was withdrawn from the receptor tube and refilled with the same volume of fresh medium. Samples were measured for DOM content spectrophotometrically at 284 nm. At the same conditions, another study was also conducted for free DOM solution for comparison. The measurements were carried out in triplicate (*n* = 3) for accuracy.

#### 2.6.5. Effect of storage on stability of DOM-loaded ethosomes

The stability of optimum formulation was studied by storage of three samples in sealed and wrapped vials at room temperature (25 °C) and at refrigerator (4 °C) for 3 months. By the end of the three months, ethosomal nanocarriers were visually inspected for any physical change followed by studying size, distribution, drug content, and *in vitro* release. Student’s *t*-test using SPSS^®^ software 22.0 (SPSS Inc., Chicago, IL) was applied to detect any statistical significance at *p*< .05.

### 2.7. Incorporation of DOM-loaded ethosomes into gels

Based on pre-formulation studies and previous reports, hydrogels were prepared using Carbopol 934 (0.5% w/w and 1% w/w) (El-Menshawe et al., 2017). Briefly, Carbopol 934 (0.5% w/w and 1% w/w) was accurately weighed and soaked in minimum amount of water under constant stirring (500 rpm) for 24 h until smooth lump-free homogenous gel bases were observed. A weighed amount of the optimized ethosomal suspension equivalent to 30 mg DOM was added to the swollen polymer that was further completed to 50 g with distilled water and stirring using homogenizer till homogenous gels were obtained (El-Leithy et al., 2010). An appropriate quantity of TEA was added drop wise to neutralize the pH 6.8 (Paliwal et al., 2019). Control gel was also prepared with plain drug (30 mg) following the same previous procedure. The composition of the prepared formulations is given in Table 3.

**Table 3.** Composition of prepared gels loaded DOM-ethosomal suspension.

Formulations code	Polymer	Polymer weight (g) (w/w) (%)	Gel load	Distilled water (g)
F11	Carbopol 934	0.5	Ethosomal suspension equivalent to 30 mg DOM	Up to 50
F12		1		Up to 50
F13		0.5	30 mg free DOM	Up to 50
F14		1		Up to 50

### 2.8. Characterization of the developed DOM-loaded ethosomal gel

#### 2.8.1. Homogeneity observations

The prepared hydrogels were visually scrutinized for their homogeneity, consistency, phase separation, and presence of any aggregates (Rao Amarachinta et al., 2021).

#### 2.8.2. pH measurement

Evaluating pH of the developed hydrogels was recorded using calibrated digital pH meter (3310, Jenway, UK) in order to determine tendency of gel for causing mucosal irritation. One gram of gel preparation was dispersed in 20 mL distilled water and the pH was measured by completely dipping of the glass electrode into the gel system (Ismail et al., 2021).

#### 2.8.3. Drug content

The prepared gels were analyzed for drug content to assure the productivity of method of preparation presented as minimal drug loss. Accurately weighed 1 g of gel was dissolved in 25 mL of methanol and stirred for 1 h till complete solubility and complete release of drug. Take 5 mL from the solution to be filtered through 0.45 mm syringe filter. One milliliter of the filtrate was diluted to 25 mL with methanol and the drug concentrations were measured spectrophotometrically at *λ*_max_ 284 nm against blank. The average of three readings was taken (Shelke & Kulkarni, 2018; Tiwari et al., 2021).

#### 2.8.4. Spreadability measurements

Spreadability assessment is essential indicator for uniform spreading of rectal hydrogels as well as its appropriate viscosity (Asad et al., 2021). Practically, 1 g of the prepared hydrogel was burdened between two glass slides of the same thickness (25 in.). Then, a certain weight (1 kg) located over the slides for 1 min; and subsequently the gel was spread out the slides. Finally, weight was removed and the diameter of the spread area in (cm) was then determined (Jaswanth Gowda et al., 2021).

#### 2.8.5. Rheological studies

The viscosity of the prepared gels was inspected using Brookfield viscometer (Brookfield viscometer, model DV-III, programmable rheometer, spindle 7, Middleboro, MA) at 25 ± 10 °C. Before measurement, 10 g of each formula was placed in a beaker and allowed to settle down for 30 min at room temperature. The spindle dipped groove in each gel without touching the bottom of the beaker and rotated at different speeds; 5, 6, 10, 12, 20, 30, 50, 60, and 100 rpm. The viscosity readings were recorded and the average of three readings was taken (Tiwari et al., 2021). The shear rate in s^−1^, the shear stress in dyne/cm^2^ and the viscosity in centipoises (cps) were determined.

#### 2.8.6. Mucoadhesive strength

The mucoadhesive strength of the gels was determined according to the method reported by Patel & Patel (2020) with some modifications (Patel & Patel, 2020). It measures the force needed for detachment of the formulation from mucous membrane of rabbit rectum in well stirred container. Briefly, equal segments of rabbit rectum were surgically apart and placed in cold saline solution. They stored separately in phosphate buffer pH 6.8 at 4 °C till use. Each segment of rectal mucous membrane was placed on each side of two glass slides and fixed by thread. A certain amount of gel was placed on one slide while the second was attached to mucosal membrane which was inverted to attach to gel. The gel hence was sandwiched between two mucous membranes for certain time to allow creation of adhesive forces between gel and mucous membrane. The weight or mass required for detachment of gel from mucous membrane and breakdown this adhesive force is measured and mucoadhesive force (dyne/cm^2^) is calculated according to this equation (Shah et al., 2011).

$$\text{Mucoadhesive strength }(\mathrm{dyne}/{\mathrm{cm}}^{2})=\frac{m\;\times \;g}{A}\times 100$$

where *m* is the weight (g) required for detachment; *g* is the acceleration due to gravity (980 cm/s^2^); *A* is the area of mucosa exposed.

The rectal mucosa was changed for each measurement and the experiment was repeated triplicate.

### 2.9. *In vivo* pharmacokinetic study

*In vivo* pharmacokinetic study was carried to estimate the rate of DOM transmucosal permeation from the optimum DOM-loaded ethosomal gel comparing to conventional DOM rectal gel. The experimental protocol was approved by the Animal Research Ethical Committee, Faculty of Pharmacy, Helwan University (code 03A2021). The *in vivo* study was performed in male Wister rats (*n* = 18) aged 2–3 months, weighing 200 g ± 10%, which were housed in metabolic cages at ambient temperature. Rats were fasted overnight (12 h) prior to formulations rectal application with free access to water. They were randomly divided into three groups (A, B, and C) with six animals in each group. Group A received saline solution (negative control) via oral gavage feeding needles (Vijayanand et al., 2016). Meanwhile, groups B and C received DOM rectal gel and DOM loaded ethosomal gel, respectively, after animals anesthetizing. The animal dose was calculated based on the human dose using the conversion factor (Reagan-Shaw et al., 2008), where amount taken from both types of gels was equivalent to 0.6 mg DOM. Blood samples (1 mL) were obtained via retro-orbital venous plexus puncture with the help of fine capillary tubes at predetermined time intervals (0.5, 1, 2, 4, 6, 9, 12, 24, 30, and 48 h post-dose). Blood samples were collected in heparinized tubes and subjected to centrifugation for 10 min at 5000 rpm at 4 °C temperature in order to separate the plasma. The separated plasma was stored at −20 °C until DOM quantification (Vijayanand et al., 2016). Separated plasma were quantified for DOM content using HPLC/MS detector (Shimadzu Auto-sampler Model SIL-20A, Quebec, Canada) adapted from reported method of DOM in plasma (Madishetti et al., 2010) with some modifications. Different pharmacokinetic parameters (*C*_max_ (ng/mL), *T*_max_ (h), and AUC_0–48_) (ng.h/mL) were obtained after rectal administration and determined by employing Kinetica^®^5 software (Thermo Fisher Scientific Inc., Waltham, MA). Both *C*_max_ (ng/mL) and *T*_max_ (h) were calculated after plasma level–time curve construction while (AUC_0–48_) (ng.h/mL) was determined utilizing linear trapezoidal method (Al-Joufi et al., 2021). Student’s *t*-test using Microsoft Office 2007, using excel package (Redmond, WA) was applied to detect any statistical significance at *p*< .05 for obtained pharmacokinetic parameters (*C*_max_ and AUC_0–48_). While non-parametric Mann–Whitney’s test using GraphPad Instat^®^ (version 3.05) (La Jolla, CA) was employed to detect any statistical significance at *p*< .05 for *T*_max_.

## 3. Results and discussion

### 3.1. Vesicular size, polydispersity, and zeta potential

The results of PS and PDI of the prepared ethosomes are presented in Table 4. The mean vesicular size of the prepared ethosomal suspensions ranged between 75.5 ± 3.63 and 344 ± 2.11 nm. The results obviously showed a statistically significant relationship between both lecithin concentrations with respect to the final suspension volume, type of additives, and vesicular size of ethosomal suspensions. At low lecithin concentration (2%), a small vesicular size was obtained (177 nm); while with rising lecithin concentration (3%) larger nanocarriers (212 nm) were shown. These results were concomitant with the results reported that increasing lecithin concentration may cause multi-laminar vesicles formation and consequences increasing in PS (Morsi et al., 2018). Moreover, these results were correlated by Prasanthi & Lakshmi results who reported that different phospholipids concentrations yield ethosomes with different size without affecting EE (Prasanthi & Lakshmi, 2012). These results were confirmed by Shen et al. who found that using lipoid S100 in preparing TEs gave smaller one that prepared by lipoid E80 (Shen et al., 2014). For the effect of incorporating of different liquid lipids (oleic acid, Labrafac^®^) to ethosomal system at both low and high lecithin concentrations (2 and 3%) produced a smaller PS. This was matched with the results found that TEs contained oleic acid showed less PS and higher elasticity than those containing sodium taurocholate (Ma et al., 2015). On the other hand, introducing different types of surfactants strongly affected PS of ethosomal suspension. The formulations containing tween 80 yielded small PS compared to formulations containing span 60 at both low and high lecithin concentration. These results may be referred to HLB value of surfactant that inversely affected PS. Thus, span 60 with the lowest HLB value resulted in the highest PS (Yeo et al., 2018).

**Table 4.** Vesicular size, PDI, zeta potential (ZP), EE%, and % drug released after 6 h (Q6h) of DOM-loaded ethosomal suspension (mean ± SD, *n* = 3).

Formulations	PS	PDI	ZP	%EE	Q6h	Desirability
F1	177.3 ± 2.96	0.39 ± 0.01	–55.3 ± 0.26	61.19 ± 4.57	40.70 ± 3.22	0.564
F2	144.3 ± 3.09	0.38 ± 0.01	–58.9 ± 0.31	67.11 ± 5.94	50.80 ± 5.46	0.667
F3	112 ± 3.3	0.32 ± 0.01	–59 ± 0.28	70.02 ± 5.52	76.30 ± 2.45	0.811
F4	75.5 ± 3.63	0.42 ± 0.01	–51.4 ± 0.35	62.20 ± 5.89	63.40 ± 7.33	0.568
F5	245 ± 5.7	0.49 ± 0.02	–39.4 ± 0.32	75.17 ± 6.66	37.30 ± 5.66	0.491
F6	212 ± 7.92	0.45 ± 0.02	–56.8 ± 0.28	75.12 ± 2.17	37.30 ± 4.32	0.500
F7	177 ± 4.35	0.4 ± 0.01	–56.8 ± 0.37	73.21 ± 8.43	41.01 ± 6.21	0.521
F8	135 ± 2.18	0.36 ± 0.01	–43 ± 0.45	77.14 ± 5.46	50.80 ± 8.23	0.537
F9	95 ± 2.23	0.27 ± 0.01	–51.7 ± 0.21	72.13 ± 7.45	45.70 ± 4.54	0.600
F10	344 ± 2.11	0.51 ± 0.01	–45.2 ± 0.23	80.14 ± 2.33	35.12 ± 6.44	0.452

PS: particle size; PDI: polydispersity index; ZP: zeta potential; Q6hrs: % cumulative drug release for 6 h.

The PDI of the dispersions lied between 0.27 ± 0.01 and 0.51 ± 0.01 as shown in Table 4. The obtained PDI values were within accepted range (<0.5) implying the uniformity of the vesicular size in the prepared suspensions with least likeliness to aggregation (Salama & Aburahma, 2016).

In literature, ZP for ethosomal system usually found between −30.6 mV and −60.8 mV. A negative sign was also observed due to the high ethanol content which provides negative charges to the polar head groups of the phospholipids making an electrostatic repulsion, which in succession, decrease the aggregation of ethosomal vesicles and improve their stability (Verma & Pathak, 2012). The results of ZP were in the range between −39.40 ± 0.32 and −59 ± 0.28 mV as depicted in Table 4. From these results, all the formulations were suggested to show high resistance against aggregation (Putri et al., 2017).

### 3.2. Entrapment efficiency of ethosomal formulations

The effect of lecithin concentration and type of additives on the %EE of DOM in ethosomal vesicles is shown in Table 4. The %EE was in the range between 61.19 ± 4.57 and 80.14 ± 2.33. Generally, the high %EE of DOM may be attributed to its high lipophilic nature (log *P* 3.9). The lecithin concentration had a statistically significant effect on %EE; as by increasing its concentration the %EE significantly increased (David et al., 2013). Moreover, the %EE was affected deeply by type of additives as it was positively influenced by incorporation of liquid lipids into the formulations (F2, F3, F7, F8, and F9). This may be owing to reducing crystallinity and increasing imperfections in the crystal lattice of lecithin which leaves enough space to accommodate drug molecules, thus, leading to improved drug loading; besides the drug solubility in liquid lipid is higher than solid lipid (Ma et al., 2015). It is well defined that surfactants with lower HLB are more lipophilic and have higher affinity to entrap lipophilic drug than those with higher HLB value which are more hydrophilic (Al-Mahallawi et al., 2015). This could explain why the highest %EE (80.14 ± 2.33%) of DOM was illustrated with F10 prepared using span 60 (HLB value 4.7). In addition, the higher %EE values of span 60 might be in relation with the thermal conductivity (TC) of the surfactant which is an important factor in justifying the effects of surfactant on %EE of lipid-based vesicles. This could suggest that, as the higher TC of surfactant, the higher the ability to form well-ordered structure and less leaky bilayer enhancing the %EE. Tween 80 also showed considerable %EE for DOM owing to its long carbon chain (El-Leithy & Abdel-Rashid, 2017).

### 3.3. Cumulative drug released for 6 h

**Figure 1.**
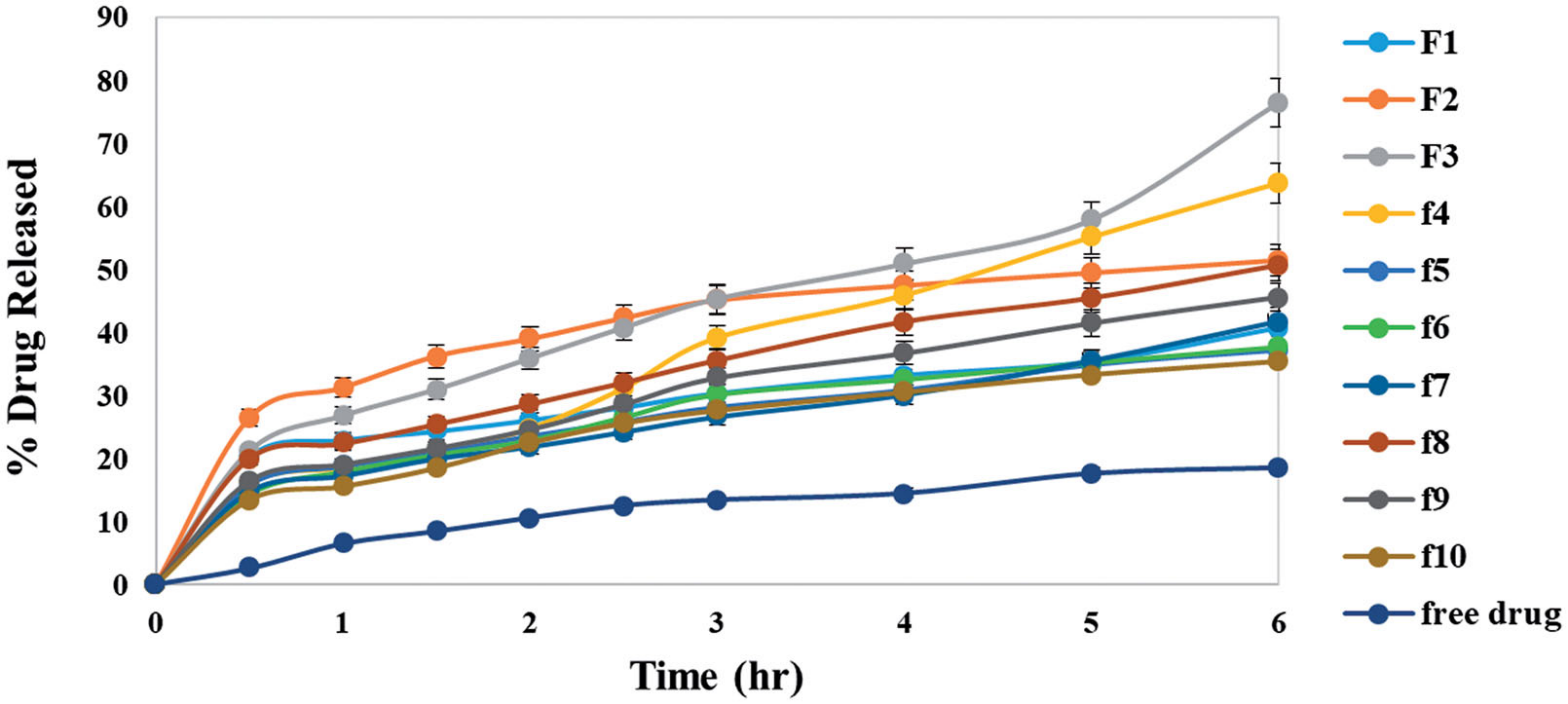
*In vitro* release profiles of DOM solution and different DOM-loaded ethosomes formulations in phosphate buffer saline (pH 6.8) at 37 °C ± 0.5 °C.

The *in vitro* release profile is a mirror image for predicting *in vivo* drug performance. The presence of ethanol in ethosomal systems distinguishes them from other lipid nano particles giving them their flexibility, easy diffusion through membranes also causes reduction in hydration layer around vesicles which facilitates drug permeation (Abdellatif et al., 2017). Figure 1 shows the release profile of DOM from all ethosomal suspension formulas as well as the free DOM which revealed that the release of DOM from all formulations was biphasic while release of free drug was gradual with only 18.4% cumulative release after 6 h. The % cumulative drug release from different formulations (Q6h) was in range of 35.12 ± 6.44 to 76.30 ± 2.45 as shown in Table 4. The results revealed that lecithin concentration had a statistically significant effect on drug release as there was a negative relationship between lecithin concentration and % drug released; by increasing concentration of lecithin % drug released decreased this owing to multi-laminar vesicles formed at the high lecithin concentration which hindered drug release. However, the addition of liquid lipids had a strong positive effect on drug release which related to their adhesion to solid lipid matrix and decreasing the diffusion path length of the lipid matrix which facilitated drug diffusion (Thatipamula et al., 2011). The formulation coded F3 prepared using 10% of lipid content as Labrafac^®^ showed the fastest release reaching 76.30 ± 2.45 after 6 h. The previous result could be justified by drug dissolution and nature of formula matrix. For surfactants as additives, they had different effects on drug release depending on their HLB as well as TC values. Hydrophilic surfactant with high HLB (tween 80) has positive effect on drug release due to its great solubilizing power on the hydrophobic drugs (Jain et al., 2003). On contrast, hydrophobic surfactants with low HLB value and high transition temperature (span 60 HLB 4.7, TC 53 °C) deeply decrease the drug release. These results were agreed with Aboud et al. who reported that low HLB/high TC surfactants may slow down the release due to the more arrangement, stability, and less leakage of the prepared vesicles (Aboud et al., 2016).

### 3.4. Response analysis according to DoE

**Figure 2.**
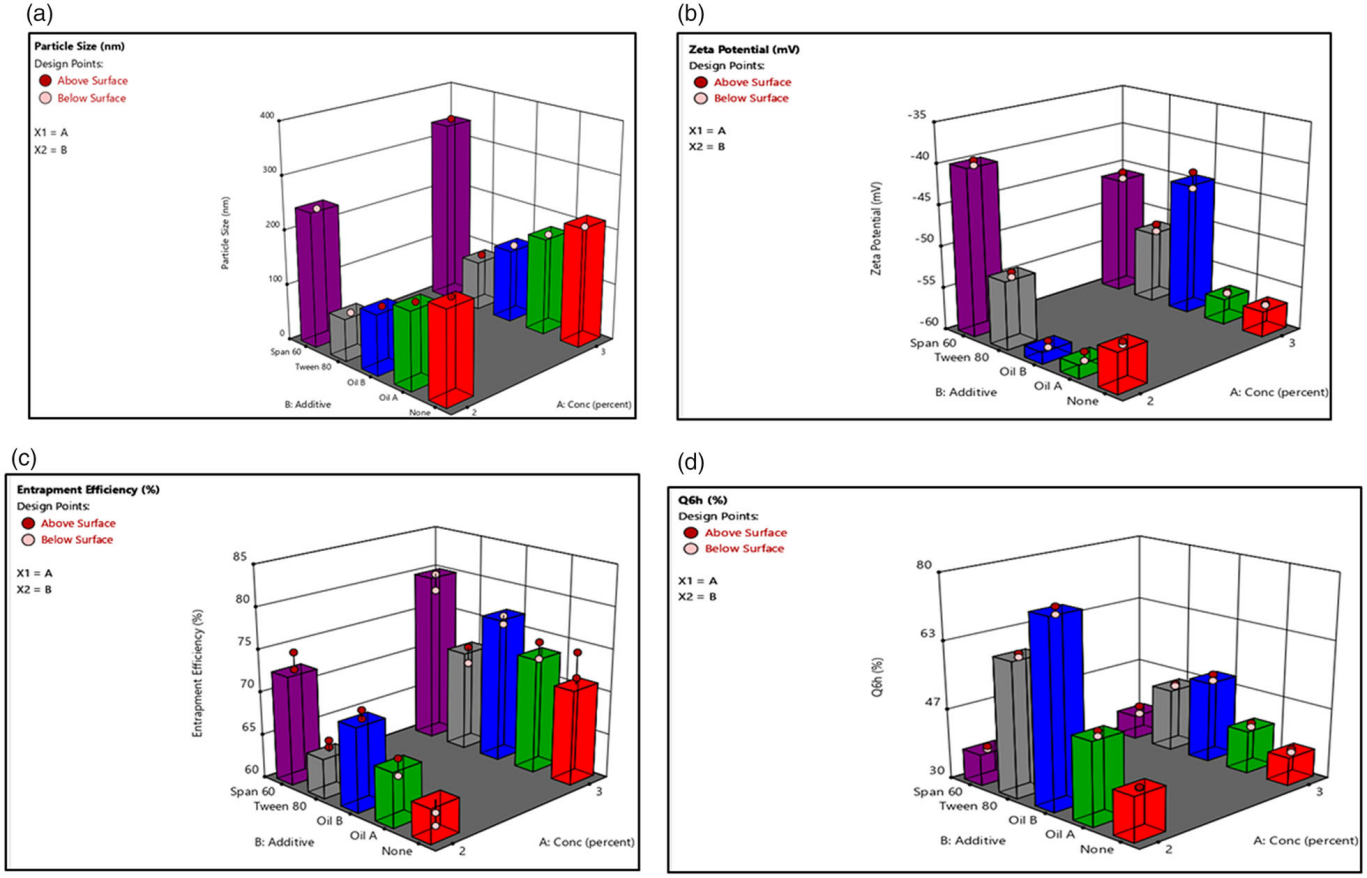
Response bar plots for the effect of lecithin concentration (%) (X1) and additives (X2) on (A) PS, (B) ZP, (C) %EE, and (D) Q6h on DOM-loaded ethosomes.

The analysis of formulations’ variables showed that there is a strong relationship between two factors (lecithin concentration (X1) and type of additives (X2) and vesicular size distribution, PDI, ZP, %EE and Q6h). Three-dimensional response bar diagrams were plotted to emphasize the effects of the interaction of these factors (X1 and X2) on vesicular size, PDI, ZP, EE%, and Q6h, respectively. Figure 2(A) illustrates that both lecithin concentration (X1) and type of additives (X2) influenced significantly (*p*<.001) the PS of the vesicles. Concerning lecithin concentration, the PS was higher at high lecithin concentration (Morsi et al., 2018). For type of additives, the PS was greater for formulations prepared using span 60 than equivalent formulations prepared using other additives (Yeo et al., 2018). On the other hand, Figure 2(B) shows that all formulations had negative surface charges ranging −39.40 ± 0.32 to −59 ± 0.28 mV suggested to show high degree of stability. Figure 2(C) shows that lecithin concentration (X1) had a positive effect on entrapment efficiency while, type of additives (X2) had a significant effect on EE% (*p*<.001). The %EE for formulations containing span 60 was higher compared to other formulations containing tween 80. Moreover, liquid lipid containing formulations had high EE% and this was in accordance with previously published results (Ma et al., 2015). On the other side, the effect of X1 and X2 on the drug release was graphically illustrated in Figure 2(D) which showed that the release of DOM was the lowest in formulations containing span 60 compared with other formulations. On contrast, the release was the highest from formulations containing Labrafac^®^ as liquid lipid as well as solubilizer. The previous results emphasized that insertion of Labrafac^®^ in composition of ethosomes has facilitated the diffusion of drug from the formulation based on high solubility and distribution of drug in the lipid matrix compared to other formulations (Aboud et al., 2016).

The analysis of the DoE results is shown in Table 5. It was observed that the predicted *R*^2^ values were in acceptable agreement with the adjusted *R*^2^ in all study responses. The adequate precision with a ratio greater than 4 was considered desirable as observed in Table 5.

**Table 5.** Output data of the (2^1^, 5^1^) full factorial analysis of the formulas, predicted and actual values for the optimum formulation (F3).

Responses	PS	PDI	ZP	%EE	Q6h
Adequate precision	55.275	43.634	38.194	15.147	65.29
Adjusted *R*^2^	0.9934	0.9912	0.9899	0.8812	0.9959
Predicted *R*^2^	0.9837	0.9782	0.977	0.7969	0.9898
Significant factors	X1, X2	X1, X2	X1, X2	X1, X2	X1, X2

### 3.5. Optimized formulation

**Figure 3.**
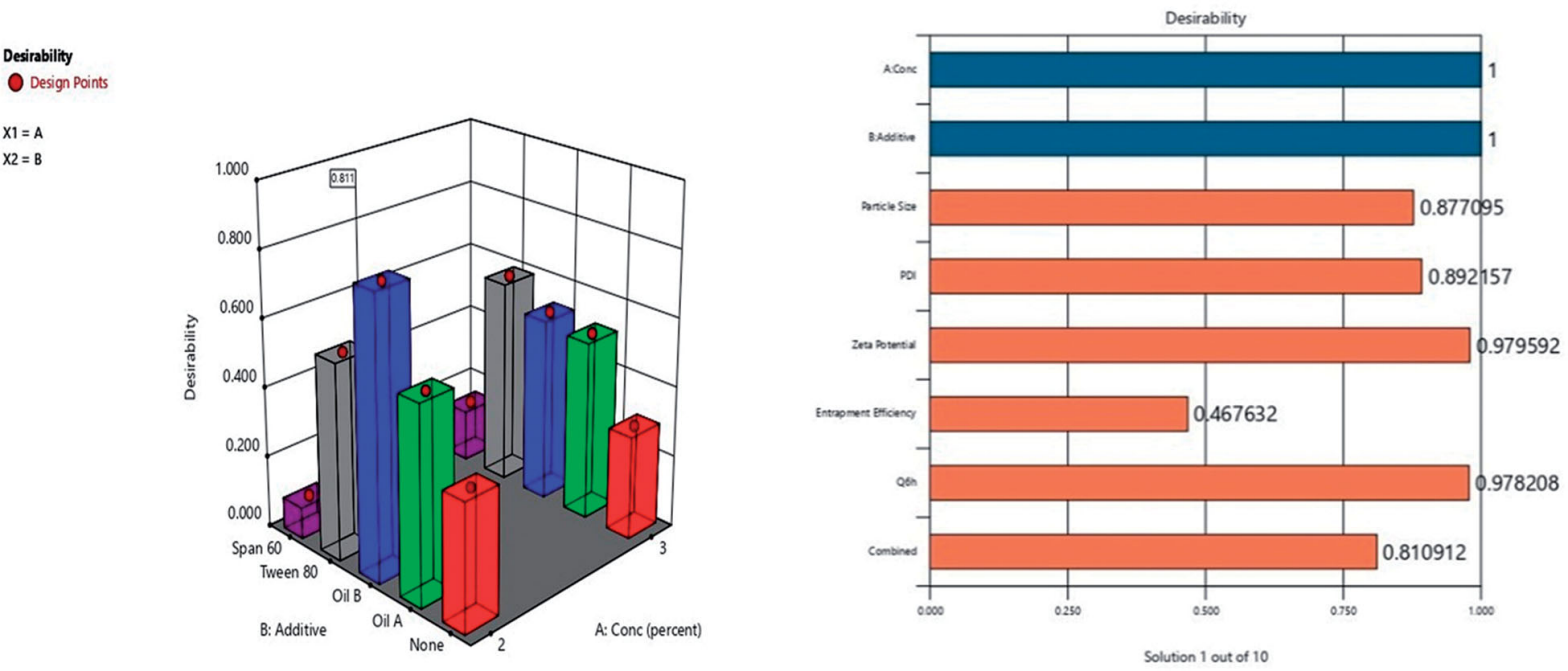
(A) Desirability 3D surface. (B) Desirability plot of the optimized formulation F3.

**Figure 4.**
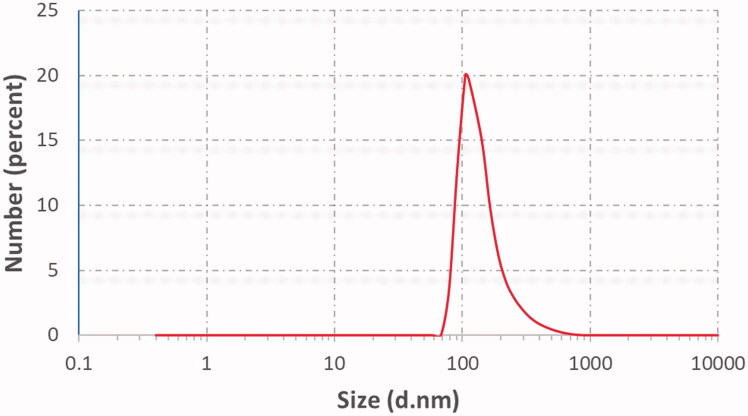
Particle size distribution of optimum formulation F3.

Hence, after performing the full factorial analysis for all formulations variables (lecithin concentration, type of additive) using Design-Expert software version 12 (Stat-Ease Inc., Minneapolis, MN) and studying their impact on different responses (vesicular size, ZP, %EE, and Q6h), the desired outcomes needed (lowest PS and PDI and highest %EE, ZP as absolute value) and Q6h were inserted to the software as shown in Table 1. The response results were inserted in the software that developed a desirability result for each formulation based upon the desired outcomes needed. The software gave the highest desirability result (0.8) to F3 which consisted of Labrafac^®^ (10%of lecithin concentration) as liquid lipid and solubilizer, lecithin 2%, ethanol 30%, PG (1 mL), and 10 mg DOM as shown in desirability graph and desirability plot in Figure 3(A,B), respectively. The figures also showed that combined effect of surfactant concentration and oil addition have caused the most significant impact on the physical characterization of the formed ethosomes. Presence of oleic acid has not only decreased vesicular size moderately but have also increased entrapment efficiency significantly. The selected optimum formulation showed %EE 70.02%, PS 112 nm, PDI 0.32, ZP −59 mV, and Q6h 76.5% as shown in Table 4 and Figure 4. Also, the software suggested the different responses results of F3. To confirm that F3 was the optimum formulation, the suggested results were statistically analyzed against the actual results of F3 and they showed non-significant difference (*p* value <.05) indicating the reproducibility and accuracy of the method of preparation (Table 6).

**Table 6.** The actual results of optimum formulation against the suggested one.

Item	Vesicular size (nm)	PDI	Zeta potential (mV)	%EE	Q6h
Actual F3 results	112	0.32	–59	70.02	76.3
Predicted F3 results	108.55	0.32	–58.6	69.885	75.4
Significance	Statistically insignificant *p* < 0.05				

PDI: polydispersity index; EE: entrapment efficiency.

### 3.6. Transmission electron microscopy

**Figure 5.**
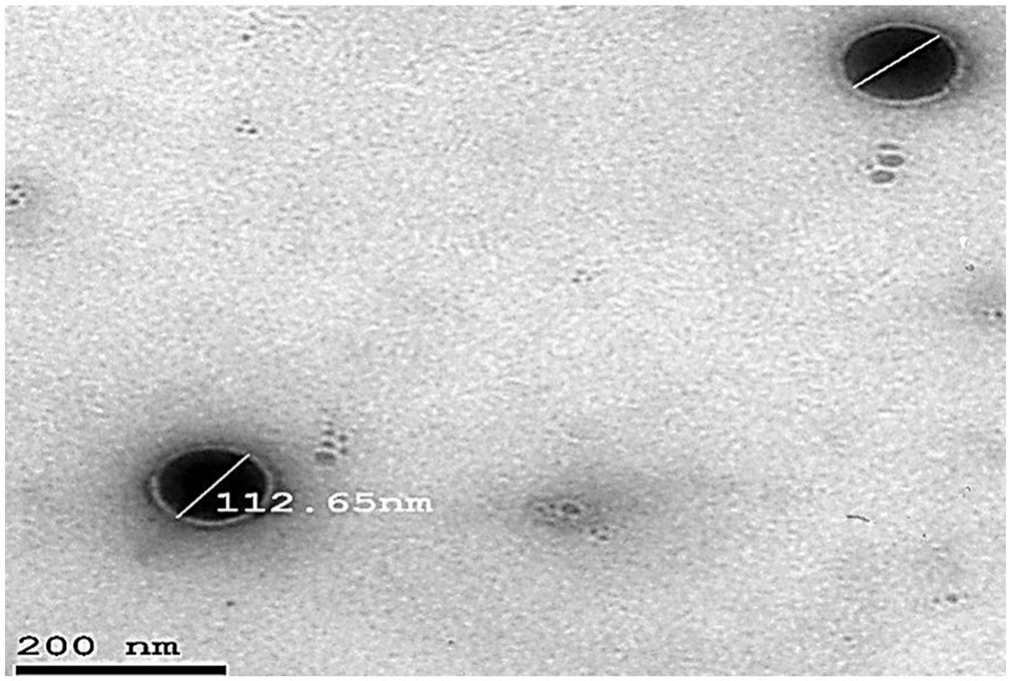
TEM image of the optimized DOM-loaded ethosomes (F3).

A representative TEM photomicrograph of optimum formulation (F3) is illustrated in Figure 5. The particles looked like a dark circular disc. The TEM examination also revealed that optimized formulation (F3) had an almost homogeneous small-sized spherical appearance with a narrow size distribution. The vesicular size shown was analogous with that determined previously by zeta sizer.

### 3.7. Differential scanning calorimetry

**Figure 6.**
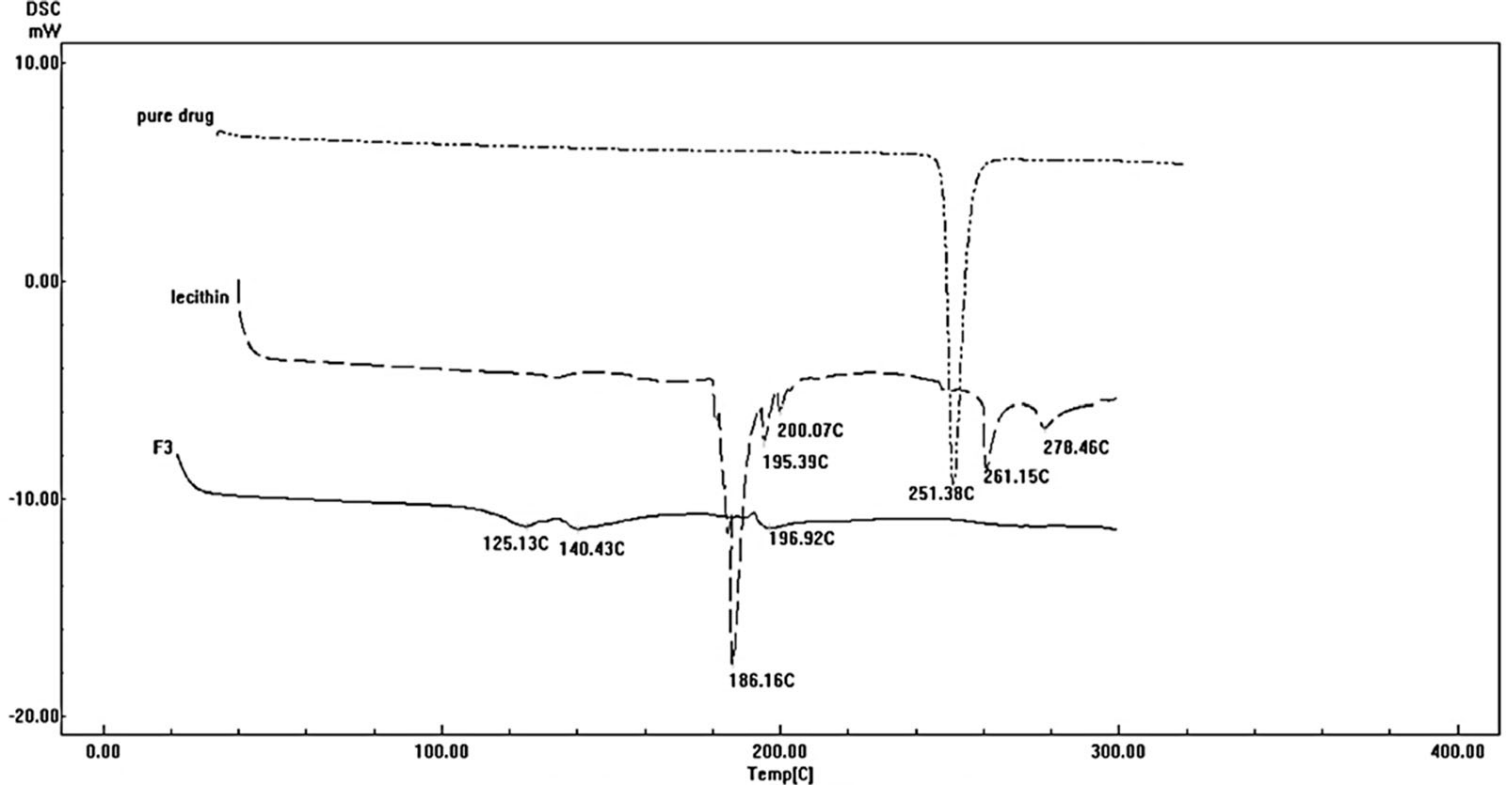
DSC thermograms of pure DOM, lecithin, and optimized DOM-loaded ethosomes (F3).

Figure 6 shows the thermograms of pure DOM, lecithin, and the optimum formulation F3. DSC thermogram of pure DOM showed a sharp endothermic peak at 251.38 °C corresponding to its melting point. The sharp endothermic peak of the DOM suggested the pure crystalline state for the drug. The thermogram of the optimum formulation (F3) showed completely disappearance of DOM endothermic peak indicating that the drug was completely solubilized inside the lipid matrix of the ethosomes. Incorporation of DOM inside the lipid matrix results in an increase in the number of defects in the lipid crystal lattice. Moreover, DOM was transformed into non crystalline state when encapsulated into ethosomes resulting in shifting of peak to lower temperature; in addition to the presence of Labrafac^®^ oil (liquid lipid) which provoked an additional shift of the melting point to lower temperature.

### 3.8. Fourier-transform infrared spectroscopy

**Figure 7.**
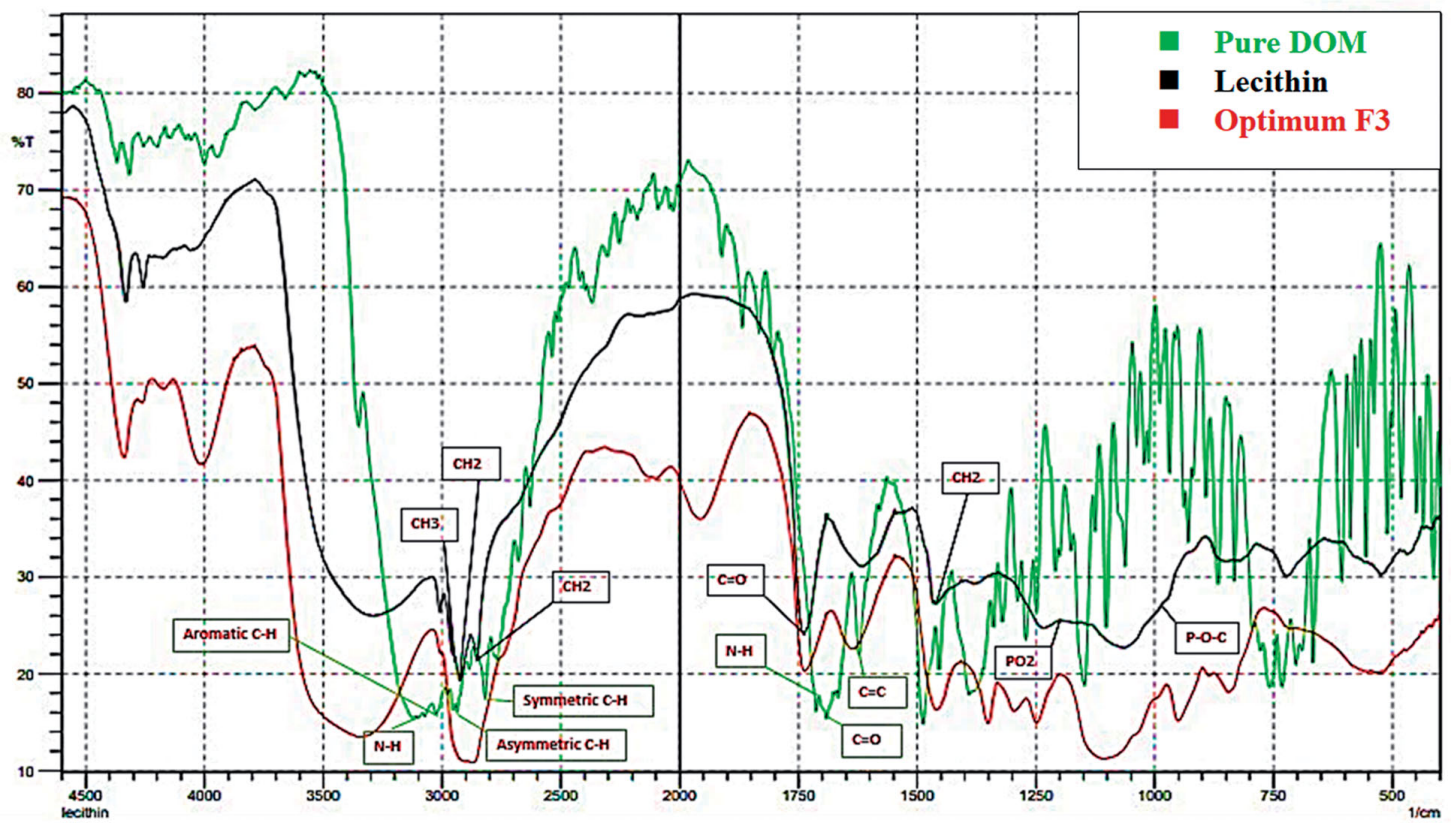
FTIR of pure DOM, lecithin, and optimum formula F3.

Figure 7 illustrates the FTIR spectra of pure DOM, PC, and F3. The FTIR spectrum of plain DOM was characterized by N–H stretching at (3122.05 cm^−1^), asymmetric C–H stretching at 2939.95 cm^−1^, symmetric C–H stretching at 2820.38 cm^−1^, N–H deformation at 1697.05 cm^−1^, aromatic C–H stretching at 3022.87 cm^−1^, C═C at 1622.02 cm^−1^ and at 1687.40 cm^−1^ owing to C═O (Enteshari & Varshosaz, 2018).

For lecithin IR spectra, there was (i) alkane bonds for symmetric CH_2_ at 2854 cm^−1^, asymmetric CH_2_ at 2928 cm^−1^, asymmetric CH_3_ stretching at 2956 cm^−1^ and 1462 cm^−1^ for CH_2_ scissoring vibrational mode; (ii) 1736 cm^−1^ corresponding to the carbonyl stretching vibration; (iii) the highly overlapped PO_2_^−^ and P–O–C infrared active vibrations in the region between 1200 and 970 cm^−1^, centered around 1061 cm^−1^ (Kuligowski et al., 2008).

The spectrum of the optimum formulation (F3) displayed no major change in the position of peaks of both pure drug and lecithin. This result revealed that there is no possible interaction between drug and excipients.

### 3.9. Effect of storage on stability

Lipid vesicular formulations including ethosomes on storage tend to some changes such as fusion or dissertation which leads to alteration in PS, PDI, ZP, and also reduction in the EE% as a result of drug leakage from the vesicles (Zeb et al., 2016). For ethosomal suspension, they were best stored under refrigerated conditions (4 °C ± 1 °C), for ensuring better physical and chemical stabilities. The PS, PDI, ZP, %EE, and Q6h of freshly prepared DOM-loaded ethosomal suspension after 3 months of storage at room temperature (25 °C) and refrigerator (4 °C) are mentioned in Table 7. These results were found to be statistically insignificant with those obtained before storage, indicating the stability of DOM-loaded ethosomal suspension. Binary ethosomes (containing PG as other alcohol) were found to be stable than classical ethosomes when stored at 4 °C (Abdulbaqi et al., 2016). Therefore, it was suggested that PG enhances ethosomes stability by increasing the viscosity and antihydrolysis property.

**Table 7.** Effect of storage on properties of selected formulation (F3).

Parameter	Fresh prepared F3	F3 after 3 months of storage at 25 °C	F3 after 3 months of storage at 4 °C
PS (nm)	112 ± 3.3	120 ± 5.4	113.5 ± 3.2
PDI	0.32 ± 0.01	0.4 ± 0.11	0.325 ± 0.04
ZP	–59 ± 0.28	–55 ± 0.79	–59 ± 0.56
%EE	70.02 ± 5.5	65.9 ± 2.30	69.88 ± 1.13
Q6h	76.30 ± 2.45	72.99 ± 4.5	76.45 ± 3.35

EE%: entrapment efficiency percentage; PDI: polydispersity index; PS: particle size; Q6h: amount of drug released after 6 h; ZP: zeta potential.

Data are presented as mean ± SD (*n* = 3).

### 3.10. *Ex vivo* transmucosal permeability

**Figure 8.**
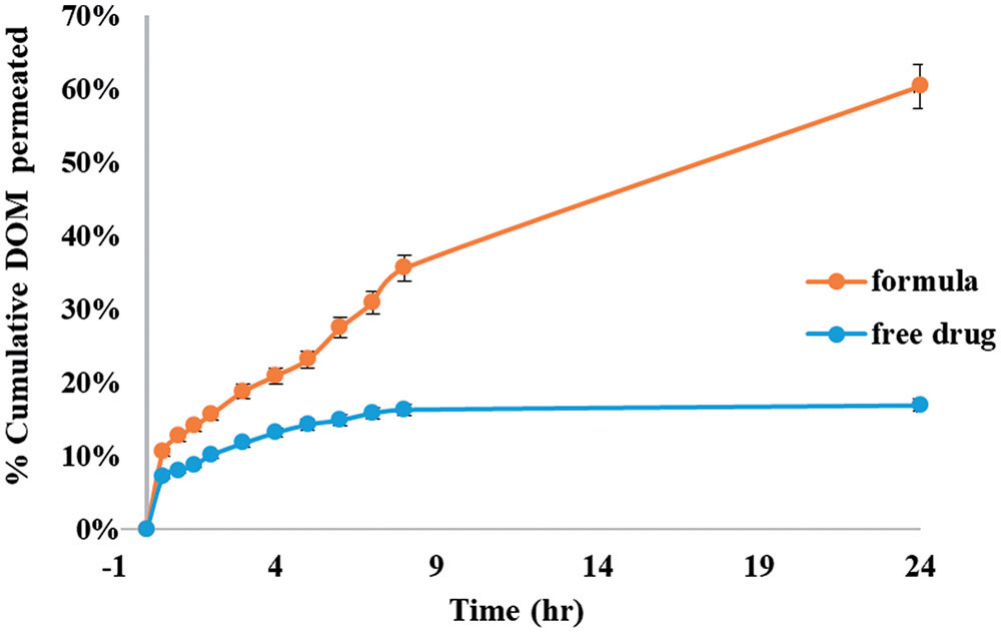
*Ex vivo* permeation profiles of DOM from prepared ethosomal suspension (F3) compared to pure drug.

The %cumulative DOM permeated through rabbit intestine mucous membrane was presented in Figure 8, showing the ability of ethosomal system in improving drug permeation through transmucosal membrane. The % cumulative DOM permeated from the optimum formulation was 60.41% in comparison to pure DOM that was about 16.90% after 24 h. The improvement in permeation confirmed the excellent deformability of the ethosomal vesicles (Verma & Pathak, 2012). The permeation power of the prepared ethosomes may be attributed to the presence of ethanol with PC that led to an increase in the vesicle flexibility and improve their ability to deform, thereby allowing them to squeeze through the mucosa (Syed et al., 2017; Pilch & Musiał, 2018). The promising results could be justified by the outstanding and unique structure of ethosomes that allows enhanced penetration of the drug through deep mucous membranes. Ethanol causes fluidity of the lipid in the cell membrane and leads to partial dissolution of the lipid intercellular matrix. Besides, under the influence of ethanol, lipid membranes of ethosomes itself become more elastic improving the ethosomes ability to release the drug in deeper mucosal layers. Experimental results highly recommend the use of ethosomal suspensions as delivery systems for DOM due to their deep effect on improving %permeation of highly hydrophobic drug (DOM) that reached up to fourfolds than free drug.

### 3.11. DOM loaded ethosomal gel

In order to expand drug permeation and pharmacological activity, the optimum ethosomal suspension was loaded into hydrogel prepared from Carbopol 934 at two concentrations (0.5% and 1%) based on screening and previously published articles which displayed that in general Carbopol 934 forms a gel at concentrations (0.5–2%) (Rathod & Mehta, 2015).

### 3.12. Characterization of DOM loaded ethosomal gel

#### 3.12.1. Homogeneity of hydrogel

The developed ethosomal gel expert good homogeneity, smoothness, and translucent appearance. They were slippery to touch with absence of any lumps indicating uniform scattering of ethosomal suspension into Carbopol gel (Ismail et al., 2021).

#### 3.12.2. pH of DOM ethosomal gels

The pH of gels is a good indicator for its irritation (Ahad et al., 2016). The pH values of developed hydrogels were in the close range of neutral pH 6.8–7.4 adjacent to rectum pH 6.8. Hence, they can avoid tissue irritation upon rectal application. Moreover, these values were sufficient to acquire a good viscosity and clarity of the gels (Dantas et al., 2016).

#### 3.12.3. Drug content

The % drug content of the prepared hydrogels was found in the range 96.2–99.3%, which revealed non-significant drug loss during the gelling process (Ansari et al., 2021).

#### 3.12.4. Spreadability

In order to meet the ideal requirements for the prepared hydrogel, good spreadability should be accomplished. Spreading values revealed behavior of gel upon application as well as affecting its therapeutic efficacy (Zakaria et al., 2016). The spreadability values of F11 ethosomal rectal gel (0.5% Carbopol) and F12 ethosomal rectal gel (1% Carbopol) were found to be 25.5 cm and 12.8 cm, respectively. These values indicated that the Carbopol provided spreadable hydrogels by shearing force of low magnitude. From the results, it was observed that spreadability of hydrogels decreased by increasing the polymer concentration. These results were matched with those obtained by Sanjana et al. (2021 ); who displayed that increasing polymer concentration results in greater restriction for the distance traveled by gel owing to the increased viscosity of the mucoadhesive polymer (Jaswanth Gowda et al., 2021). In general, the spreadability values indicated the easy application of hydrogels upon a slight amount of shear confirming it is retaining for an excellent contact time at the site of application without leakage.

#### 3.12.5. Rheological behavior of the prepared gels

The consistency and thickness of hydrogel formulation were assessed by viscosity measurements, which express its resistance to flow (Afzal et al., 2022). Viscosity and rheological behavior of hydrogels affected its mucoadhesion, playing vital role in drug release from the vehicle and hence significantly affects bioavailability. Moreover, it affords good expectations for gels mucoadhesive strength as well as its residence time (El-Leithy et al., 2010). The viscosity of formulations prepared at room temperature should be in an optimum range hence facilitated its administration. The hydrogels prepared by 0.5% Carbopol (F11 and F13) showed viscosity 7021 and 17,828 cps, respectively, while those which prepared by1% Carbopol (F12 and F14) had viscosity of 9955 and 15,900 cps, respectively. The results obviously showed the positive effect of Carbopol concentration on viscosity of gel preparations. Furthermore, the results could be correlated to the mechanism of gelling occur by Carbopol 934; which bind with the solvent strongly, establishing cross-linking and thus entrap water inside holding solvent molecules to form a firm structure resistant to certain forces. Accordingly, the higher level of Carbopol 934, the higher viscosity of the prepared hydrogels (Dawood et al., 2019).

**Figure 9.**
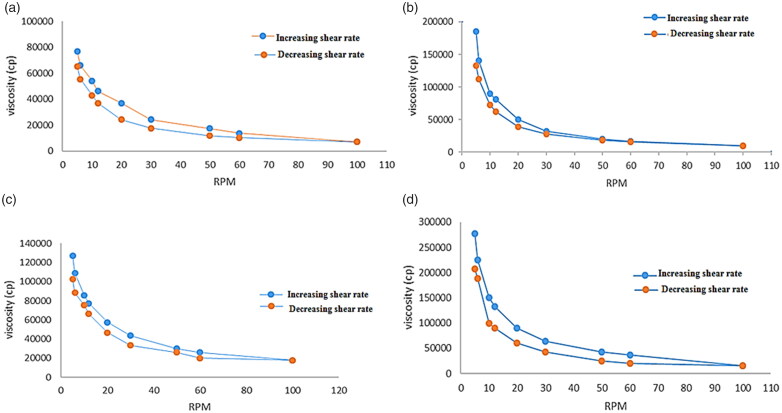
Rheological behavior of (a) (F11) ethosomal rectal gel (0.5% Carbopol), (b) (F12) ethosomal rectal gel (1% Carbopol), (c) (F13) free drug rectal gel 0.5% Carbopol, and (d) (F14) free drug rectal gel (1% Carbopol).

Figure 9(a–d) displays the rheograms of the prepared hydrogels constructed between viscosity (cps) and shearing rate (rpm) in order to study their flow behaviors. Rheological behavior of the prepared hydrogels was obeying shear thinning pseudo-plastic (non-Newtonian) behavior, where the viscosity declined by increasing shear rate displaying steady state behavior at greater levels of shear rates (Asad et al., 2021). This was favored due to the low flow resistance upon application at high shear conditions, hence lasting without drain at the application site (Dantas et al., 2016). These results could be explained by the arrangement and motion of the polymer molecules when shear rates increased; they were disheveled, elongated and oriented themselves in the direction of flow decreasing shear resistance, and hence resulting decrease in the viscosity (Panwar et al., 2020). This can be beneficial for the hydrogel since they become easier to spread and less painful upon application.

From the results, all hydrogels possessed thixotropic behaviors with different degrees which is desirable for pharmaceutical preparations as well as patient compliance; ensuring delivering thick product in thin, easily applicable and spreadable form (Dawood et al., 2019). As thixotropic behavior (self-healing), where the reversible destroyed gel during continuous shear and the recovery at static conditions took limited time (time dependent flow). Therefore, it is considered one of the most remarkable assets for the prepared hydrogels (Sugioka et al., 2021).

#### 3.12.6. Mucoadhesive strength

Using mucoadhesive polymers reinforce the gel strength and its bio-adhesion to rectal mucous membrane preventing the hydrogels from reaching the end of the colon. Mucoadhesion relies mainly on the polymers functional groups (e.g. hydroxyl, ether oxygen, and amine) which liable for formation adhesive force with mucous membrane. Therefore, the degree of mucoadhesion depends on concentration, nature and composition of bioadhesive polymers where all increased the gel strength in a concentration-dependent manner. Moreover, the mucoadhesive strength is affected by the contact time with the membrane, degree of swelling of the polymer, hydration, and the type of biological membranes (Li et al., 2017). The mucoadhesive forces for 0.5% Carbopol gel containing F3 and free drug were 3512.39 ± 4.5 and 3489.97 ± 5.7, respectively, while 1% Carbopol gel containing F3 and free drug were 4215.62 ± 7.2 and 4145.78 ± 6.5, respectively. The obtained results revealed that all formulations had high adhesive property due to using Carbopol 934 as mucoadhesive polymer which increased by increasing polymer concentration from 0.5% w/w to 1% w/w. This could be attributed to the optimum hydration and swelling of Carbopol 934 which hydrates and swells in aqueous phase due to hydrogen bonding of its carboxylic groups with sugar residues of oligosaccharide chains of mucus glycoproteins as well as the electrostatic repulsion after neutralization reaction (Begum et al., 2021). The prospective hydration of Carbopol 934 as well as its hydrogen bonding interaction with mucin licenses ideal contact with the mucus upon application with merged mucoadhesion.

### 3.13. *In vivo* pharmacokinetic study

**Figure 10.**
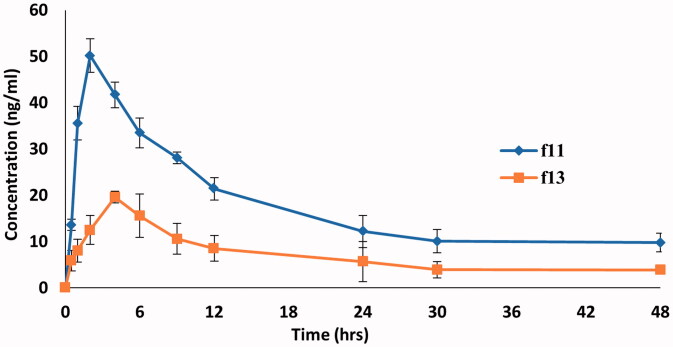
Mean DOM plasma concentration-time curves in rats after administration of a single dose (3.1 mg/kg) of DOM-loaded ethosomal gel (F11) and pure DOM gel (F13).

The LC–MS/MS method was used to fix the pharmacokinetic parameters of DOM in rat plasma as a mirror to the *in vivo* behavior of DOM ethosomal gel compared to the DOM as a reference. This method was validated and confirmed good linearity from 0.72–79.85 ng/mL. As depicted in Figure 10 and Table 8, the DOM ethosomal gel (F11) showed a significantly higher *C*_max_ of 50.2 ± 3.65 ng/mL and *T*_max_ of 2 h compared to pure DOM gel (F13) with a *C*_max_ of 19.6 ± 4.65 ng/mL and *T*_max_ of 4 h. Moreover, the AUC_0–48_ of DOM ethosomal gel (F11) was 839.95 ± 21.2 ng.h/mL compared to pure gel with AUC_0–48_ of 335.25 ± 28.55 ng.h/mL. The results also showed that relative bioavailability of the prepared DOM-loaded ethosomal gel was nearly 2.5 folds that of traditional Dom gel, meanwhile the mean residence time of traditional DOM gel (16 h) was nearly half that of ethosomal gel (33 h). From the results, it was observed that by trapping DOM into sub-micron vesicles (ethosomes), the *C*_max_ and AUC_0–48_ of DOM in the rat plasma were raised by twofold compared to pure DOM gel owing to increase solubility and dissolution rate. Our findings were parallel to previous report results which have revealed that the bioavailability of drugs could be improved by entrapping them in submicron vesicles (Ramadon et al., 2017). The results were well matched with the results of *ex vivo* transmucosal permeation study where the ethosomal suspension increase % of drug permeated up to 4 folds than pure DOM suspension owing to ethosomes deformability and flexibility which boost permeation and penetration deeply through mucosal layers (El-Menshawe et al., 2017). Another possible reason may be owing to Carbopol 934 which modulates the tight junctions hence enhances paracellular drug transport (Balakrishnan et al., 2015). Thus, bioavailability could significantly increase when compared to pure DOM gel (*p*<.05).

**Table 8.** Mean pharmacokinetics parameters of DOM following single rectal administration for DOM-loaded ethosomal gel (F11) and pure DOM gel (F13).

Pharmacokinetic parameters	DOM-loaded ethosomal gel (F11)	Pure DOM gel (F13)
*C*_max_ (ng/mL)	50.2 ± 3.65	19.6 ± 4.65
*T*_max_ (h)	2	4
AUC_0–48_ (ng.h/mL)	839.95 ± 21.2	335.25 ± 28.55
%Relative bioavailability	250%	100%
Elimination rate constant (ke)	0.0314 h^−1^	0.061 h^−1^
Mean residence time	33 h	16 h
Half-life (*t*_1/2_)	22 h	11.3 h

Data are represented as mean ± SD (*n* = 3).

All the experiments in the study were repeated at least three times and the data were expressed as the mean ± standard deviation (SD). Statistical analysis of data was performed using ANOVA. As presented in Table 8, statistical analysis of the pharmacokinetic parameters revealed that there was a significant difference (*p*<.05) between values of *C*_max_, *T*_max_, and AUC_0–48_ of both DOM-loaded ethosomal gel (F11) and pure DOM gel (F13).

## 4. Conclusions

The study proved that DOM-loaded ethosomes could be simply prepared in satisfactory and cost effective way. Ten formulations were successfully formulated and characterized using 5^1^, 2^1^ full factorial design. The optimum formulation was selected according to the high desirability values obtained by the design. The ethosomal suspension attained obvious increase in the DOM permeation (3.57-folds) compared to free drug. The *in vivo* pharmacokinetic parameters of DOM ethosomes loaded in hydrogels were nearly doubled compared to conventional gel. Thus, ethosomal gels can be a potential approach for enhancing rectal transmucosal drug delivery.

## Data Availability

All data and materials are available.

## References

[CIT0001] Abdellatif AAH, Tawfeek HM. (2016). Transfersomal nanoparticles for enhanced transdermal delivery of clindamycin. AAPS PharmSciTech 17:1477–74. 26511937 10.1208/s12249-015-0441-7

[CIT0002] Abdellatif MM, Khalil IA, Khalil MAF. (2017). Sertaconazole nitrate loaded nanovesicular systems for targeting skin fungal infection: in-vitro, ex-vivo and in-vivo evaluation. Int J Pharm 527:1–11. 28522423 10.1016/j.ijpharm.2017.05.029

[CIT0003] Abdulbaqi IM, Darwis Y, Abdul Karim Khan N, et al. (2016). Ethosomal nanocarriers: the impact of constituents and formulation techniques on ethosomal properties, in vivo studies, and clinical trials. Int J Nanomed 25:2279–304. 10.2147/IJN.S105016 PMC4887071 27307730

[CIT0004] Aboud HM, Ali AA, El-Menshawe SF, Elbary AA. (2016). Nanotransfersomes of carvedilol for intranasal delivery: formulation, characterization and in vivo evaluation. Drug Deliv 23:2471–81. 25715807 10.3109/10717544.2015.1013587

[CIT0005] Afzal S, Zahid M, Rehan ZA, et al. (2022). Preparation and evaluation of polymer-based ultrasound gel and its application in ultrasonography. Gels 8:42. 35049577 10.3390/gels8010042 PMC8774352

[CIT0006] Ahad A, Aqil M, Kohli K, et al. (2016). The ameliorated longevity and pharmacokinetics of valsartan released from a gel system of ultradeformable vesicles. Artif Cells Nanomed Biotechnol 44:1457–63. 25953248 10.3109/21691401.2015.1041638

[CIT0007] Albash R, Abdelbary AA, Refai H, El-Nabarawi MA. (2019). Use of transethosomes for enhancing the transdermal delivery of olmesartan medoxomil: in vitro, ex vivo, and in vivo evaluation. Int J Nanomedicine 14:1953–68. 30936696 10.2147/IJN.S196771 PMC6421897

[CIT0008] Al-Joufi F, Elmowafy M, Alruwaili NK, et al. (2021). Mucoadhesive in situ rectal gel loaded with rifampicin: strategy to improve bioavailability and alleviate liver toxicity. Pharmaceutics 13:336. 33807729 10.3390/pharmaceutics13030336 PMC8001001

[CIT0009] Al-Mahallawi AM, Abdelbary AA, Aburahma MH. (2015). Investigating the potential of employing bilosomes as a novel vesicular carrier for transdermal delivery of tenoxicam. Int J Pharm 485:329–40. 25796122 10.1016/j.ijpharm.2015.03.033

[CIT0010] Ansari SA, Qadir A, Warsi MH, et al. (2021). Ethosomes-based gel formulation of karanjin for treatment of acne vulgaris: in vitro investigations and preclinical assessment. 3 Biotech 11:456. 10.1007/s13205-021-02978-3 PMC8497663 34631355

[CIT0011] Asad MI, Khan D, Ur Rehman A, et al. (2021). Development and in vitro/in vivo evaluation of pH-sensitive polymeric nanoparticles loaded hydrogel for the management of psoriasis. Nanomaterials 11:3433–3. 34947782 10.3390/nano11123433 PMC8705938

[CIT0012] Athukuri BL, Neerati P. (2017). Enhanced oral bioavailability of domperidone with piperine in male Wistar rats: involvement of CYP3A1 and P-gp inhibition. J Pharm Pharm Sci 20:28–37. 28459658 10.18433/J3MK72

[CIT0013] Balakrishnan P, Park E, Song C, et al. (2015). Carbopol-incorporated thermoreversible gel for intranasal drug delivery. Molecules 20:4124–35. 25749681 10.3390/molecules20034124 PMC6272239

[CIT0014] Begum MY, Alqahtani A, Ghazwani M, et al. (2021). Preparation of Carbopol 934 based ketorolac tromethamine buccal mucoadhesive film: in vitro, ex vivo, and in vivo assessments. Int J Polym Sci 2021:1–11.

[CIT0015] Bendas ER, Tadros MI. (2007). Enhanced transdermal delivery of salbutamol sulfate via ethosomes. AAPS PharmSciTech 8:213–20. 10.1208/pt0804107 PMC2750693 18181528

[CIT0016] Cevc G, Blume G. (1992). Lipid vesicles penetrate into intact skin owing to the transdermal osmotic gradients and hydration force. Biochim Biophys Acta 1104:226–32. 1550849 10.1016/0005-2736(92)90154-e

[CIT0017] Dantas MGB, Reis SAGB, Damasceno CMD, et al. (2016). Development and evaluation of stability of a gel formulation containing the monoterpene borneol. ScientificWorldJournal 2016:7394685. 27247965 10.1155/2016/7394685 PMC4876256

[CIT0018] David SR, Hui MS, Pin CF. (2013). Formulation and in vitro evaluation of ethosomes as vesicular carrier for enhanced topical delivery of isotretinoin. Int J Drug Deliv 5:28–34.

[CIT0019] Dawood NM, Jassim Z, Mowafaq G, Zaki H. (2019). Studying the effect of different gelling agent on the preparation and characterization of metronidazole as topical emulgel. Asian J Pharm Clin Res.

[CIT0020] El-Menshawe SFE, Kharshoum RM, El Sisi AM. (2017). Preparation and optimization of buccal propranolol hydrochloride nanoethosomal gel: a novel approach for enhancement of bioavailability. J Nanomed Nanotechnol 8:435.

[CIT0021] El Sayed MMA, Abdallah OY, Naggar VF, Khalafallah NM. (2006). Deformable liposomes and ethosomes: mechanism of enhanced skin delivery. Int J Pharm 322:60–6. 16806755 10.1016/j.ijpharm.2006.05.027

[CIT0022] El-Leithy ES, Shaker DS, Ghorab MK, Abdel-Rashid RS. (2010). Evaluation of mucoadhesive hydrogels loaded with diclofenac sodium-chitosan microspheres for rectal administration. AAPS PharmSciTech 11:1695–702. 21108027 10.1208/s12249-010-9544-3 PMC3011065

[CIT0023] El-Leithy ES, Abdel-Rashid RS. (2017). Lipid nanocarriers for tamoxifen citrate/coenzyme Q10 dual delivery. J Drug Deliv Sci Technol 41:235–50.

[CIT0024] El-Shenawy AA, Abdelhafez WA, Ismail A. (2019). Formulation and characterization of nanosized ethosomal formulations of antigout model drug (febuxostat) prepared by cold method: in vitro/ex vivo and in vivo assessment. AAPS PharmSciTech 21:1–31. 31858305 10.1208/s12249-019-1556-z

[CIT0025] Enteshari S, Varshosaz J. (2018). Solubility enhancement of domperidone by solvent change in situ micronization technique. Adv Biomed Res 7:109. 30069440 10.4103/abr.abr_219_17 PMC6050975

[CIT0026] Ferrier J. (2014). Domperidone as an unintended antipsychotic. Can Pharm J 147:76–83. 10.1177/1715163514521969 PMC3962062 24660005

[CIT0027] Hesketh PJ, Kris MG, Basch E, et al. (2017). Antiemetics: American Society of Clinical Oncology Clinical Practice Guideline Update. J Clin Oncol 35:3240–61. 28759346 10.1200/JCO.2017.74.4789

[CIT0028] Honeywell-Nguyen PL, Frederik PM, Bomans PHH, et al. (2002). Transdermal delivery of pergolide from surfactant-based elastic and rigid vesicles: characterization and in vitro transport studies. Pharm Res 19:991–7. 12180552 10.1023/a:1016466406176

[CIT0029] Ismail TA, Shehata TM, Mohamed DI, et al. (2021). Quality by design for development, optimization and characterization of brucine ethosomal gel for skin cancer delivery. Molecules 26:3454. 34200144 10.3390/molecules26113454 PMC8201187

[CIT0030] Jain S, Jain P, Umamaheshwari RB, Jain NK. (2003). Transfersomes—a novel vesicular carrier for enhanced transdermal delivery: development, characterization, and performance evaluation. Drug Dev Ind Pharm 29:1013–26. 14606665 10.1081/ddc-120025458

[CIT0031] Jaswanth Gowda BH, Adinaryan S, Ahmed MG. (2021). Preparation and evaluation of in-situ gels containing hydrocortisone for the treatment of aphthous ulcer. J Oral Biol Craniofac Res 11:269–76. 33717865 10.1016/j.jobcr.2021.02.001 PMC7920855

[CIT0032] Kadam VS, Bharkad VB, Shete GA. (2017). Formulation and evaluation of fast dissolving oral film of metoclopramide HCl. World J Pharm Pharm Sci 6:2052–66.

[CIT0033] Kuligowski J, Quintás G, Garrigues S, de la Guardia M. (2008). Determination of lecithin and soybean oil in dietary supplements using partial least squares-Fourier transform infrared spectroscopy. Talanta 77:229–34. 18804625 10.1016/j.talanta.2008.06.029

[CIT0034] Li X, Ye Z, Wang J, et al. (2017). Mucoadhesive buccal films of tramadol for effective pain management. Rev Bras Anestesiol 67:231–7. 27899200 10.1016/j.bjan.2016.10.006

[CIT0035] Ma M, Wang J, Guo F. (2015). Development of nanovesicular systems for dermal imiquimod delivery: physicochemical characterization and in vitro/in vivo evaluation. J Mater Sci Mater Med 26:6–191. 10.1007/s10856-015-5524-1 25989936

[CIT0036] Madishetti SK, Palem CR, Gannu R, et al. (2010). Development of domperidone bilayered matrix type transdermal patches: physicochemical, in vitro and ex vivo characterization. Daru 18:221–9. 22615620 PMC3304359

[CIT0037] Matthews M, Glackin M, Hughes C, Rogers KMA. (2015). Who accesses complementary therapies and why? An evaluation of a cancer care service. Complement Ther Clin Pract 21:19–25. 25544064 10.1016/j.ctcp.2014.09.005

[CIT0038] Morsi NM, Aboelwafa AA, Dawoud MHS. (2018). Enhancement of the bioavailability of an antihypertensive drug by transdermal protransfersomal system: formulation and in vivo study. J Liposome Res 28:137–48. 28264602 10.1080/08982104.2017.1295989

[CIT0039] Mukherjee S, Ray S, Thakur RS. (2009). Design and evaluation of itraconazole loaded solid lipid nanoparticulate system for improving the antifungal therapy. Pak J Pharm Sci 22:131–8. 19339221

[CIT0040] Paliwal S, Tilak A, Sharma J, et al. (2019). Flurbiprofen loaded ethosomes – transdermal delivery of anti-inflammatory effect in rat model. Lipids Health Dis 18:133. 31170970 10.1186/s12944-019-1064-x PMC6554971

[CIT0041] Panwar P, Michael P, Devlin M, Martini M. (2020). Critical shear rate of polymer-enhanced hydraulic fluids. Lubricants 8:102.

[CIT0042] Patel AP, Patel JK. (2020). Mucoadhesive in-situ gel formulation for vaginal delivery of tenofovir disoproxil fumarate. Indian J Pharm Educ Res 54:963–70.

[CIT0043] Patil HG, Tiwari RV, Repka MA, Singh KK. (2016). Formulation and development of orodispersible sustained release tablet of domperidone. Drug Dev Ind Pharm 42:906–15. 26472165 10.3109/03639045.2015.1088864

[CIT0044] Phillips RS, Friend AJ, Gibson F. (2016). Antiemetic medication for prevention and treatment of chemotherapy-induced nausea and vomiting in childhood. Cochrane Database Syst Rev 2:CD007786. 26836199 10.1002/14651858.CD007786.pub3 PMC7073407

[CIT0045] Pilch E, Musiał W. (2018). Liposomes with an ethanol fraction as an application for drug delivery. Int J Mol Sci 19:3806. 10.3390/ijms19123806 PMC6320757 30501085

[CIT0046] Prasanthi D, Lakshmi PK. (2012). Development of ethosomes with Taguchi robust design based studies for transdermal delivery of alfuzosin hydrochloride. Int Curr Pharm J 1:370–5.

[CIT0047] Putri DCA, Dwiastuti R, Marchaban M, Nugroho AK. (2017). Optimization of mixing temperature and sonication duration in liposome preparation. J Pharm Sci Community 14:79–85.

[CIT0048] Radtke M, Souto EB, Muller RH. (2005). Nanostructured lipid carriers: a novel generation of solid lipid drug carrier. Pharm Technol Eur 17:45–50.

[CIT0049] Ramadon D, Anwar E, Harahap Y. (2017). In vitro penetration and bioavailability of novel transdermal quercetin-loaded ethosomal gel. Indian J Pharm Sci 79:948–56.

[CIT0050] Rao Amarachinta P, Sharma G, Samed N, et al. (2021). Central composite design for the development of carvedilol-loaded transdermal ethosomal hydrogel for extended and enhanced anti-hypertensive effect. J Nanobiotechnol 19:100. 10.1186/s12951-021-00833-4 PMC8035747 33836744

[CIT0051] Rathod H, Mehta D. (2015). A review on pharmaceutical gel. Int J Pharm Sci 1:33–47.

[CIT0052] Reagan-Shaw S, Nihal M, Ahmad N. (2008). Dose translation from animal to human studies revisited. FASEB J 22:659–61. 17942826 10.1096/fj.07-9574LSF

[CIT0053] Safwat S, Ishak RAH, Hathout RM, Mortada ND. (2017). Nanostructured lipid carriers loaded with simvastatin: effect of PEG/glycerides on characterization, stability, cellular uptake efficiency and in vitro cytotoxicity. Drug Dev Ind Pharm 43:1112–25. 28276784 10.1080/03639045.2017.1293681

[CIT0054] Salama AH, Aburahma MH. (2016). Ufasomes nano-vesicles-based lyophilized platforms for intranasal delivery of cinnarizine: preparation, optimization, ex-vivo histopathological safety assessment and mucosal confocal imaging. Pharm Dev Technol 21:706–15. 25996631 10.3109/10837450.2015.1048553

[CIT0055] Satija A, Ahmed SM, Gupta R, et al. (2014). Breast cancer pain management—a review of current & novel therapies. Indian J Med Res 139:216–25. 24718395 PMC4001332

[CIT0056] Sayed SI, Mohammed A, Mohammed A, et al. (2015). Formulation by design-based proniosome for accentuated transdermal delivery of risperidone: in vitro characterization and in vivo pharmacokinetic study. Drug Deliv 22:1059–70. 24471715 10.3109/10717544.2013.870260

[CIT0057] Sguizzato M, Mariani P, Spinozzi F, et al. (2020). Ethosomes for coenzyme q10 cutaneous administration: from design to 3D skin tissue evaluation. Antioxidants 9:485. 10.3390/antiox9060485 PMC7346166 32503293

[CIT0058] Shah RA, Mehta MR, Patel DM, Patel CN. (2011). Design and optimization of mucoadhesive nasal in situ gel containing sodium cromoglycate using factorial design. Asian J Pharm 5:65–74.

[CIT0059] Shelke O, Kulkarni A. (2018). Formulation, development and evaluation of nano ethosomal gel of tramadol hydrochloride. J Nanomed Nanotechnol 9:514.

[CIT0060] Shen L-N, Zhang Y-T, Wang Q, et al. (2014). Enhanced in vitro and in vivo skin deposition of apigenin delivered using ethosomes. Int J Pharm 460:280–8. 24269286 10.1016/j.ijpharm.2013.11.017

[CIT0061] Song CK, Balakrishnan P, Shim C-K, et al. (2012). A novel vesicular carrier, transethosome, for enhanced skin delivery of voriconazole: characterization and in vitro/in vivo evaluation. Colloids Surf B Biointerfaces 92:299–304. 22205066 10.1016/j.colsurfb.2011.12.004

[CIT0062] Sugioka Y, Nakamura J, Ohtsuki C, Sugawara-Narutaki A. (2021). Thixotropic hydrogels composed of self-assembled nanofibers of double-hydrophobic elastin-like block polypeptides. Int J Mol Sci 22:4104. 33921095 10.3390/ijms22084104 PMC8071462

[CIT0063] Syed SI, Abdul A, Mohamed A, et al. (2017). Formulation by design based risperidone nano soft lipid vesicle as a new strategy for enhanced transdermal drug delivery: in-vitro characterization, and in-vivo appraisal. Mater Sci Eng C Mater Biol Appl 75:1198–205. 28415407 10.1016/j.msec.2017.02.149

[CIT0064] Thatipamula RP, Palem CR, Gannu R. (2011). Formulation and in vitro characterization of domperidone loaded solid lipid nanoparticles and nanostructured lipid carriers. Daru 19:23–32. 22615636 PMC3232070

[CIT0065] Tiwari A, Bag P, Sarkar M, et al. (2021). Formulation, validation and evaluation studies on metaxalone and diclofenac potassium topical gel. Environ Anal Health Toxicol 36:e2021001. 33499562 10.5620/eaht.2021001 PMC8207004

[CIT0066] Touitou E, Godin B, Dayan N, et al. (2001). Intracellular delivery mediated by an ethosomal carrier. Biomaterials 22:3053–9. 11575480 10.1016/s0142-9612(01)00052-7

[CIT0067] Verma P, Pathak K. (2012). Nanosized ethanolic vesicles loaded with econazole nitrate for the treatment of deep fungal infections through topical gel formulation. Nanomedicine 8:489–96. 21839053 10.1016/j.nano.2011.07.004

[CIT0068] Vijayanand P, Patil J, Reddy MV. (2016). Formulation, characterization and in vivo evaluation of novel edible dosage form containing nebivolol HCl. Braz J Pharm Sci 52:179–90.

[CIT0069] Yeo L, Olusanya T, Chaw C, Elkordy A. (2018). Brief effect of a small hydrophobic drug (cinnarizine) on the physicochemical characterisation of niosomes produced by thin-film hydration and microfluidic methods. Pharmaceutics 10:185. 10.3390/pharmaceutics10040185 PMC6321096 30322124

[CIT0070] Zakaria AS, Afifi SA, Elkhodairy KA. (2016). Newly developed topical cefotaxime sodium hydrogels: antibacterial activity and in vivo evaluation. Biomed Res Int 2016:6525163. 27314033 10.1155/2016/6525163 PMC4895050

[CIT0071] Zeb A, Qureshi OS, Kim H-S, et al. (2016). Improved skin permeation of methotrexate via nanosized ultradeformable liposomes. Int J Nanomedicine 11:3813–24. 27540293 10.2147/IJN.S109565 PMC4982511

[CIT0072] Zhou Y, Wei Y, Liu H, et al. (2010). Preparation and in vitro evaluation of ethosomal total alkaloids of *Sophora alopecuroides* loaded by a transmembrane pH-gradient method. AAPS PharmSciTech 11:1350–8. 20740333 10.1208/s12249-010-9509-6 PMC2974122

